# Pregabalin administration in patients with fibromyalgia: a Bayesian network meta-analysis

**DOI:** 10.1038/s41598-022-16146-x

**Published:** 2022-07-15

**Authors:** Filippo Migliorini, Nicola Maffulli, Matthias Knobe, Giacomo Tenze, Ali Aljalloud, Giorgia Colarossi

**Affiliations:** 1 Department of Orthopaedic, Trauma, and Reconstructive Surgery, RWTH Aachen University Clinic, Pauwelsstraße 30, 52074 Aachen, Germany; 2 Department of Medicine, Surgery and Dentistry, University of Salerno, Via S. Allende, 84081 Baronissi, SA Italy; 3 School of Pharmacy and Bioengineering, Keele University School of Medicine, Thornburrow Drive, Stoke on Trent, England; 4 Barts and the London School of Medicine and Dentistry, Centre for Sports and Exercise Medicine, Mile End Hospital, Queen Mary University of London, 275 Bancroft Road, London, E1 4DG England; 5 Department of Orthopedics and Trauma Surgery, Lucerne Cantonal Hospital, Lucerne, Switzerland; 6 Department of Emergency Urgency Intensive Care Unit, University Clinic Tor Vergata, 00133 Rome, Italy; 7 Department of Cardiothoracic Surgery, RWTH Aachen University Clinic, 52074 Aachen, Germany

**Keywords:** Drug safety, Drug screening, Pharmaceutics, Medical research, Rheumatology

## Abstract

Several studies investigated the effectiveness and the safety of different doses of pregabalin in fibromyalgia. However, the optimal protocol remains controversial. A Bayesian network meta-analysis comparing 300, 450, and 600 mg/daily of pregabalin for fibromyalgia was conducted. The literature search was conducted in January 2022. All the double-blind randomised clinical trials comparing two or more dose protocols of pregabalin for fibromyalgia were accessed. Studies enrolling less than 50 patients were not eligible, nor were those with a length of follow-up shorter than eight weeks. The outcomes of interests were: Fibromyalgia Impact Questionnaire (FIQ), sleep quality, and adverse events. The network meta-analyses were performed using the routine for Bayesian hierarchical random-effects model analysis, with log odd ratio (LOR) and standardized mean difference (SMD) effect measure. Data from 4693 patients (mean age 48.5 years) were retrieved. 93.1% (4370 of 4693 patients) were women. The median follow-up was 14.8 weeks. Pregabalin 450 mg/daily resulted in greater reduction in Fibromyalgia Impact Questionnaire (SMD − 1.83). Pregabalin 600 demonstrated the greatest sleep quality (SMD 0.15). Pregabalin 300 mg/daily evidenced the lowest rate of adverse events (LOR 0.12). The dose of pregabalin must be customised according to patients’ characteristics and main symptoms.

## Introduction

Approximately 2% of the adult population suffer from fibromyalgia^1^. Chronic widespread pain is the major symptom of fibromyalgia, along with fatigue, depression, and sleep disorders^2^. Although the aetiology of fibromyalgia has not yet been fully clarified, alterations of pain processing and regulation pathways have been documented^3,4^. Neurotransmitters abnormalities, including glutamatergic neurotransmission, have been demonstrated^5^. Moreover, affected patients evidenced high levels of glutamate in specific brain areas involved in pain control, such as in the insula lobe^6^. Gabapentanoids reduce the glutamatergic activity and modulate the increased functional connectivity between brain areas in chronic pain^7^. Among gabapentinoids, pregabalin, a gamma-aminobutyric acid (GABA) analogue, inhibits calcium channels, reducing presynaptic neurotransmitters release and postsynaptic excitability^8,9^. Pregabalin is primarily an anticonvulsant; however, given its effects on central pain modulation, it was approved for patients with fibromyalgia^10,11^. Several studies investigated the effectiveness and the safety of pregabalin^12–15^. However, the optimal dose of pregabalin for the management of fibromyalgia remains controversial^16–21^. Therefore, a Bayesian network meta-analysis was conducted to compare the use of 300, 450 and 600 mg/daily of pregabalin for fibromyalgia. Different to the conventional direct head to head meta-analyses, Bayesian network meta-analyses allow to compare of two or more interventions, allowing indirect comparisons based on strict logical deduction^22^. The present study focused on patient reported outcome measures (PROMS) and the rate of adverse effects.

## Methods

### Search strategy

This Bayesian network meta-analysis was conducted according to the PRISMA extension statement for reporting of systematic reviews incorporating network meta-analyses of health care interventions^23^. The PICOTD framework was preliminary pointed out:

- P (Problem): Fibromyalgia;
- I (Intervention): Pregabalin;
- C (Comparison): 300- 450- 600 mg/daily;
- O (Outcomes): clinical scores, adverse events;
- T (Timing): ≥ 8 weeks follow-up;
- D (Design): double-blinded randomized clinical trials.

### Data source and extraction

The literature search was conducted independently by two authors (F.M. and G.C.) in January 2022. PubMed, Google scholar, Embase, and Scopus databases were accessed. The following search strategy weas used in all database: fibromyalgia [All Fields] AND, pain [All Fields], FIQ [All Fields], fibromyalgia impact questionnaire [All Fields], sleep quality [All Fields], OR management [All Fields], pharmacological [All Fields], pharmacology [All Fields], treatment [All Fields], COMBINED WITH pregabalin [All Fields], 300 [All Fields], 450 [All Fields], 600 [All Fields], gaba [All Fields], doses [All Fields], mg [All Fields], daily [All Fields], complications [All Fields], adverse events [All Fields], drug [All Fields]. The Resulting articles were screened by the same two authors. If title and abstract matched the topic, the full-text of the articles of interest was accessed. A cross-reference of the bibliography of the full-text articles was also performed. Disagreements were solved by a third senior author (N.M.).

### Eligibility criteria

All the double-blind randomized controlled trials (RCTs) comparing two or more dose protocols of pregabalin in patients with fibromyalgia were accessed. Given the authors language abilities, articles in English, German, Italian, French and Spanish were eligible. Only prospective studies with at least level II of evidence, according to Oxford Centre of Evidence-Based Medicine (OCEBM)^24^, were considered. Only studies that clearly stated the doses of pregabalin were considered for inclusion. Compounds other than pregabalin were considered as control group. To ensure the reliability of the data to be collected, studies enrolling less than 50 patients were excluded, as were those with a length of follow-up shorter than eight weeks. Reviews, letters, expert opinion, editorials were not considered. Studies combining pregabalin with other compounds were not considered. Missing quantitative data under the outcomes of interest warranted the exclusion from the present study.

### Outcomes of interest

Data extraction was performed by two authors (F.M. and G.C.). Study generalities (author, year, and journal) and patient baseline demographic information were extracted (number of samples, mean age, and sex). The following data were extracted: Fibromyalgia Impact Questionnaire (FIQ) total score, mean sleep quality, and adverse events. The mean sleep quality is a numeric rating scale, in which 0 represent the best and 10 the worst possible sleep. The outcomes of interests were to compare 300; 450; 600 mg/daily of pregabalin at last follow-up.

### Methodology quality assessment

The methodological quality assessment was performed by two authors (F.M. and G.C.) using the bias graph tool of the Review Manager Software (The Nordic Cochrane Collaboration, Copenhagen). The following risk of bias were evaluated: selection, detection, performance, reporting, attrition and other source of bias.

### Statistical analysis

The statistical analysis was performed by the main author (F.M.). The Shapiro–Wilk test was performed to investigate data distribution for baseline assessment. Parametric data were assessed using mean and standard deviation. Non-parametric data were assessed using median and interquartile range. The analysis of variance (ANOVA) and the Kruskal–Wallis test were respectively performed, with values of *P* > 0.1 considered satisfactory. The network meta-analyses were performed through the STATA Software/MP Version 16 (StataCorporation, College Station, Texas, USA) using the routine for Bayesian hierarchical random-effects model analysis. The inverse variance method was used for all the comparisons. The Log odd ratio (LOR) effect measures was used for dichotomous variables, while the standardized mean difference (SMD) for the continuous variables. The overall inconsistency was evaluated through the equation for global linearity via the Wald test. If *P* value > 0.1, the null hypothesis could not be rejected, and the consistency assumption is accepted at the overall level of each treatment. Both confidence (CI) and percentile (PrI) intervals were set at 95%. Edge plots, interval plots, funnel plots and ranking plots were obtained and evaluated.

### Ethical approval

This study complies with ethical standards.

## Results

### Search result

**Figure 1:**
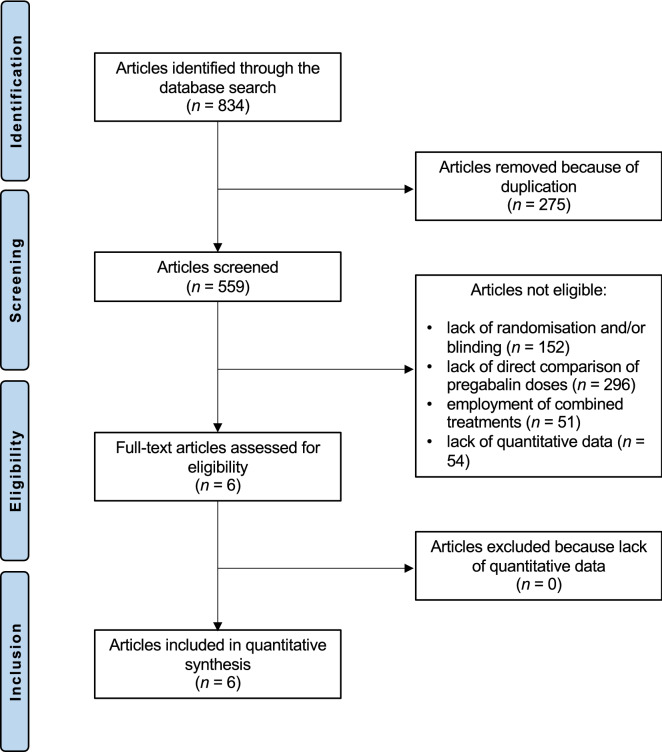
Flow chart of the literature search.

The literature search resulted in 834 articles. Of them, 275 were duplicates. A further 553 articles were not eligible because of lack of randomisation and/or blinding (N = 152), lack of direct comparison of pregabalin doses (N = 296), employment of combined treatments (N = 51), lack of quantitative data (N = 54). Finally, six double-blind RCTs were included (Fig. 1).

### Methodological quality assessment

**Figure 2:**
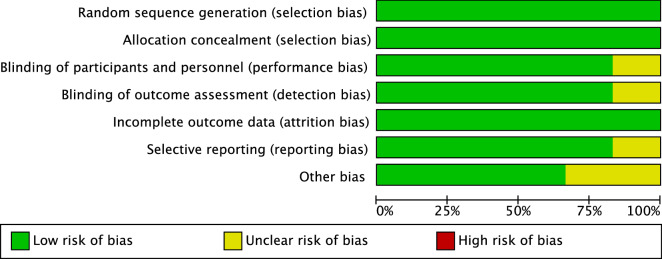
Methodological quality assessment.

The random sequence generation and allocation concealment were adequately described and unbiased, leading to a low risk of selection bias. Given the double-blinded nature of the study design of the included studies, the risk of performance and detection were low. The risk of attrition bias of incomplete outcome data was low, as was the risk of reporting bias. The reviewers found no other risk of bias which may exert an influence in the validity of the present study. Concluding, the risk of publication bias was very low, attesting to the present study an optimal methodological quality assessment (Fig. 2).

### Patient demographics

Data of 4693 patients were retrieved. 93.1% (4370 of 4693 patients) were women. The mean age of the patients was 48.5 ± 0.9 years. Only two studies^16,18^ reported data concerning the mean BMI, which was 30.0 ± 0.8 kg/m^2^. The median follow-up was 14.8 ± 5.9 weeks. The ANOVA test found good baseline comparability in terms of age, BMI, and gender (*P* > 0.1). Generalities and demographic of the study are shown in Table 1.

**Table 1:** Generalities and patient baseline of the included studies.

References	Journal	Follow-up (weeks)	Dosis	Patients (*n*)	Mean Age	Women (%)
Argoff et al*.*^16^	*Pain Med*	14	300 mg/d	453	47.8	
			450 mg/d	459	47.3	
			Control Group	457	46.9	
Arnold et al.^17^	*J Pain*	14	300 mg/d	183		95
			450 mg/d	190		96
			600 mg /d	188		95
			Control Group	184		92
Crofford et al.^21^	*Arthritis Rheum*	8	Control Group	132	48	96
			300 mg/d	134	47.7	90
			450 mg/d	132	48.9	90
			Control Group	131	49.7	91
Crofford et al.^19^	*Pain*	26	Control Group	287	49.6	94
			300 mg/d	63	49.6	95
			450 mg/d	73	49	95
			600 mg/d	143	48.4	91
Maese et al.^18^	*J Rheumatol*	13	300 mg/d	185	50.1	94
			450 mg/d	183	47.7	92
			600 mg/d	190	48.7	95
			Control Group	190	48.6	96
Pauer et al.^20^	*J Rheumatol*	14	300 mg/d	184	48.4	91
			450 mg/d	182	48	93
			600 mg/d	186	49.6	91
			Control Group	184	48.1	91

### Outcomes of interest

**Figure 3:**
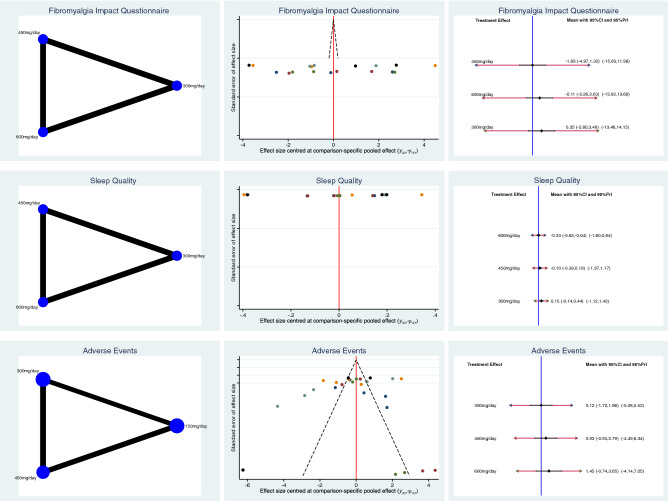
Results of network comparisons.

Pregabalin 450 mg/daily reported the greatest FIQ improvement (SMD − 1.83; 95% CI − 4.97 to 1.32). Pregabalin 600 demonstrated the greatest sleep quality (SMD 0.15; 95% CI − 0.14 to 0.44). Pregabalin 300 mg/daily evidenced the lowest rate of adverse events (LOR 0.12; 95% CI − 1.72 to 1.96). The equation for global linearity found no statistically significant inconsistency (*P* > 0.1). These results are shown in greater detail in Fig. 3.

## Discussion

According to the present Bayesian network meta-analysis, daily administration of 450 mg of pregabalin resulted in the greatest FIQ, while 600 mg/daily was the most effective dose improve sleep quality in fibromyalgia. Patient treated with pregabalin 300 mg/daily experienced the lowest rate of adverse events.

The sleep quality was directly proportional to dose escalation. Given the high prevalence and impact, sleep disorders are a key component of fibromyalgia, and patients evidence delayed sleep onset, more frequent arousals and decreased sleep efficiency compared with healthy controls^25^. Polysomnography demonstrated an alpha delta sleep pattern during non-REM phases^26,27^. Administration of 150 to 450 mg/daily of pregabalin for a month improved total sleep time and quality, latency to persistent sleep, the number of awakenings after sleep onset, and the amount of slow-wave sleep relative to placebo^28^. Previous placebo-controlled trials of pregabalin for the treatment of fibromyalgia showed that pregabalin improved daily sleep quality diary (DSQD) and Medical Outcomes Study-Sleep Scale (MOS-SS) scores at doses of 300, 450 and 600 mg/d^17,18,20,21,29,30^. This improvement was evident from the second week, and was sustained throughout the administration period. High association between pain relief and improvement in sleep quality was demonstrated for all doses of pregabalin^17,18,30^. As expected, the rate of adverse event was inversely proportional to the dose escalation. The interval plots reported minimal difference in the effect. Most of CI are overlapping, and the analyses of the FIQ and adverse events revealed wide CI. Moreover, the funnel plots, especially in the analysis of FIQ and sleep quality were well proportioned with a symmetrical distribution of the referral points, which indicates low data dispersion and reliable results. The funnel plot of the adverse events demonstrated greater heterogeneous distribution; however, this endpoint included a wide range of possible complications, from both pharmacological management and natural disease progression. The most commonly reported adverse events included dizziness, headache, and fatigue nausea, blurred vision, and dry mouth and sleep disorders.

The management of fibromyalgia is complex^31^. The available guidelines recommend a combined approach of pharmacological and non-pharmacological therapy^32^. Among the pharmacological therapies, pregabalin, duloxetine, and milnacipran have been approved by FDA for fibromyalgia^33^. Clair et al.^14^ collected data from five double-blind RCTs concerning the use of pregabalin for fibromyalgia. They evidenced the superiority of pregabalin in improving pain and sleep scores compared to placebo^14^. In the double-blind placebo-controlled RCT conducted by Arnold et al.^17^, 750 patients were allocated to receive pregabalin 300, 450, or 600 mg/daily or placebo. They found that the rate of 30% pain relief was 42% (76 of 183 patients), 50% (94 of 190 patients), and 48% (88 of 188 patients), respectively^17^. The rate of 50% responders was 24% (44 of 183 patients), 27% (52 of 190 patients), and 30% (57 of 188 patients), respectively^17^. Moreover, similarly to the results of the present study, they found greater FIQ improvement with 450 and 600 mg/daily compared to 300 mg/daily^17^. We were not able to identify a previous Bayesian network meta-analysis which compared the administration of different doses of pregabalin for fibromyalgia. The findings of previous head to head meta-analysis were similar to those inferred by the present study. Choy et al.^34^ in a meta-analysis found greater FIQ and sleep quality with 450 and 600 mg/daily compared to 300 mg/daily. Similarly, the current study evidenced better sleep quality in patients who take 600 and 450 mg/daily of pregabalin compared to 300 mg/daily. Moore et al.^35^ reported no difference between the efficacy of 450 and 600 mg/daily in 30% and 50% pain relief. Likewise, the intake of 300, 450, and 600 mg of pregabalin has similar potential in pain reduction^36^. Also, 150 mg/daily pregabalin promoted similar efficacy and tolerability to placebo^36^. A Bayesian network meta-analysis, not focusing exclusively on pregabalin, found that 300 mg of pregabalin was superior to 150 mg in pain relief^37^. A meta-analysis on 21 RCTs investigated several pharmacological compounds, and compared them to placebo^38^. The number of patients who achieved 30% and 50% pain relief was greater for pregabalin 450 mg/daily than 300 mg/daily. Straube et al.^29^ performed a meta-analysis on five RCTs (3808 patients) concerning the administration of pregabalin 150, 300, 450, and 600 mg/daily. 300 to 600 mg/daily produced pain relief in 30% to 50%, while 150 mg/daily demonstrated limited efficacy in 50% pain relief^29^. Similar to the results of the current study, 450 mg/daily of pregabalin demonstrated a greater impact on FIQ total score than 600 mg; the latter resulted more effective to improve sleep quality^29^.

The long-term safety and tolerability of pregabalin have been demonstrated in previous studies^39^. Somnolence, dizziness, dry mouth and peripheral oedema are the most frequent adverse events occurring in patients receiving pregabalin^40^. The meta-analysis by Haeuser et al.^36^ reported the adverse effects of different doses of pregabalin. The rate of study discontinuation from adverse events was greater for pregabalin 450 and 600 mg/daily than 300 mg/daily. In a double-blind, placebo-controlled RCT (747 patients)^20^, the occurrence of adverse events was dose-related. These findings agree with those of the present study: the administration of 300 mg/daily of pregabalin resulted in a lower rate of adverse events.

This study has several limitations. Although the current literature includes several studies investigating the potential of pregabalin in fibromyalgia, according to the inclusion criteria, we were able to identify only six RCTs that directly compared different dose regimes. Despite the high quality of the included studies, the relatively small number of patients available for analysis represent an important limitation. Studies with a follow-up shorter than eight weeks were not included for analysis. Moreover, the impact of the follow-up duration on the treatment response was also not investigated. Short follow-up may not be reliable to evaluate the effectiveness and safety of pharmacological management. Fibromyalgia is a chronic condition, and its management requires long term polytherapy. The investigation of the effect of pharmacological therapies should last longer, following the course of the conditon. The current evidence would benefit of longer observational studies. Given the lack and homogeneity of available data in the current literature, it was not possible to evaluate the full impact of pregabalin therapy on pain relief. There were not enough data on pregabalin 150 mg/daily administration to allow further considerations. However, prior studies stated that the efficacy of pregabalin 150 mg/daily was comparable to placebo administration^29,36,37^. Even though several PROMs have been proposed to investigate the therapy efficacy in fibromyalgia^12,41–44^, given the lack of data of the included studies, no further score was reliably available. Considering these limitations, the results of the present study should be considered cautiously. Future studies including larger populations should investigate long term administration of pregabalin in patients with fibromyalgia.

## Conclusion

A daily dose of 450 mg of pregabalin resulted most effective in improving FIQ total score, while 600 mg/daily resulted in greater sleep quality. The rate of adverse events was lower for 300 mg/daily than for greater doses. The dose of pregabalin must be customised according to the patient’s characteristics. It is likely that the response to pregabalin is genetically determined. To our knowledge, no such studies have been performed, and, together with better understanding of the underlying condition, they should form the basis of future studies to show better personalisation of drug therapy in fibromyalgia patients.

## Data Availability

The datasets generated during and/or analysed during the current study are available throughout the manuscript.
